# State and Local Syringe Distribution Laws and the Presence of Syringe Services Programs

**DOI:** 10.1001/jamahealthforum.2026.3730

**Published:** 2026-10-23

**Authors:** Samantha M. Doonan, Spruha Joshi, Corey S. Davis, Sugy Choi, Katherine Wheeler-Martin, Pooja Shah, Jaskiran Dhinsa, Moumita Chowdhury, Samrachana Adhikari, Ashleigh Dennis, Charles DiMaggio, Magdalena Cerdá

**Affiliations:** 1 Department of Population Health, New York University Grossman School of Medicine; 2 University of Michigan School of Public Health, Ann Arbor; 3 Harm Reduction Legal Project, Network for Public Health Law, Edina, Minnesota; 4 Editorial Fellow, *JAMA Health Forum*; 5 Department of Surgery, New York University Grossman School of Medicine

## Abstract

**Importance:**

Syringe services programs (SSPs) are an evidence-based intervention to reduce bloodborne disease transmission and other risks related to injection drug use; however, SSP access is uneven. State and local syringe distribution laws may influence variation in the presence and number of SSPs across local jurisdictions.

**Objective:**

To assess the association between state and local syringe distribution laws and presence of publicly listed SSPs in a sample of US cities.

**Design, Setting, and Participants:**

This cross-sectional study linked state and local laws with information on publicly reported SSPs in 2024 for the largest US municipalities and census-designated places (localities). The outcomes of policy exposure in SSP presence and total number of SSP locations among localities with at least 1 SSP were estimated using a 2-part negative binomial hurdle model, adjusting for state and local policy and demographic and health characteristics. The study team collected the legal data from January 11, 2023, through May 7, 2024.

**Exposure:**

State and local syringe distribution policy environment as of December 31, 2022, categorized from most restrictive (state prohibition) to least restrictive (neither state nor locality prohibits syringe distribution).

**Main Outcomes and Measures:**

Publicly reported SSP presence (binary) and total count of SSP locations within each locality as of February 2024.

**Results:**

In this study using data from 482 US localities, there was at least 1 SSP in 181 (37.6%) localities. Localities with the least restrictive legal environment had increased odds of SSP presence (adjusted odds ratio, 16.3; 95% CI, 6.0-44.1) compared with those with the most restrictive legal environment. Among localities with 1 or more SSP, localities in the least restrictive legal environment or a legal environment that exempted SSPs from a syringe distribution prohibition had increased rates of SSPs, relative to the most restrictive legal environment (rate ratio for least restrictive policy environment, 2.7; 95% CI, 1.1-6.4; rate ratio for legal environment that exempts SSPs from prohibition, 2.4; 95% CI, 1.1-5.6). Overall, policies that exempted SSPs from syringe distribution prohibitions and those with no syringe distribution prohibitions had the highest estimated SSP counts (1.9 SSPs; 95% CI, 1.5-2.4; 1.5 SSPs; 95% CI, 1.1-1.9, respectively).

**Conclusions and Relevance:**

This study found that state and local syringe distribution laws were associated with publicly listed SSP presence. Legal environments that prohibit or restrict syringe distribution may limit the presence and total number of SSPs, highlighting the role of policy context in shaping access to harm-reduction services.

## Key Points

**Question:**

What is the association between state and local syringe distribution laws and the presence and total number of syringe services programs (SSPs) in US cities?

**Findings:**

In this cross-sectional study using data from 482 US localities, 38% of the largest cities had at least 1 publicly listed SSP in 2024. Localities with no state or local syringe distribution prohibitions or where SSPs were exempt from those prohibitions had substantially higher odds of any publicly listed SSP presence and had greater counts of SSPs in places with at least 1 SSP, compared with localities in states that prohibited syringe distribution.

**Meaning:**

Findings of this study suggest that state and local syringe distribution laws are important structural factors associated with SSP availability.

## Teaser

This cross-sectional study reports on the association between US state and local syringe distribution laws and the presence and number of syringe service programs.

## Introduction

Opioid use disorder, overdose, HIV, and hepatitis C virus (HCV) operate as a syndemic in the US.^1^ In 2024, more than 80 000 people died of a drug-related overdose.^2^ There were an estimated 67 000 acute HCV cases in 2022,^3^ and more than 38 000 people were newly diagnosed with HIV.^4^ People who inject drugs are particularly impacted.^3,4^ Syringe services programs (SSPs) are an evidence-based strategy to reduce harms related to injection drug use.^5^

SSPs are community-based programs that provide a range of health and social services designed to reduce harms experienced by people who use drugs, particularly those related to injection drug use.^1,5,6,7^ SSPs play an important role in reducing bloodborne disease transmission among people who inject drugs.^6,8,9,10,11,12^ They do so, in part, through education, testing and treating HIV and HCV,^13^ and through the provision of sterile injection equipment, which leads to lower levels of needle and syringe sharing among participants.^14,15,16^ SSPs have also adapted to meet community needs in the contemporary overdose crisis. In 2021, more than 90% of SSPs provided overdose education and naloxone to participants, and more than 80% provided fentanyl test strips.^17^ Further, more than 30% of SSPs provided access to medication treatment for opioid use disorder on site.^18^ However, access to SSP services is uneven across the US, and SSPs are absent in many areas.^19^

Given the benefits of SSPs, it is critical to pinpoint the structural factors that can improve SSP availability. SSP implementation and operation are constrained by a variety of state and local laws, many of which either prohibit free syringe distribution outright, restrict SSP operations, or create legal ambiguity.^20,21,22^ Local approval processes may also inhibit SSP operations and growth.^21^ Further, there is evidence that the state law environment around SSPs influences downstream health outcomes of people who use drugs,^23,24,25^ suggesting that these laws are important levers to shape population health outcomes.^26^ Despite the importance of both state and local law to SSP implementation and operation, prior studies have focused primarily on state laws.^23,24,25,26^ This study addressed that gap by estimating whether both the state (or District of Columbia) and local syringe distribution policy environment were associated with the presence of publicly listed SSPs (any and total number) in a large sample of US cities in 2024.

## Methods

### Sample

This cross-sectional study leveraged a dataset compiled by study team members (C.S.D., A.D., K.W.M., P.S., J.D., M.C., S.J., and S.M.D.) to characterize state and local laws for 482 of the largest US municipalities and census-designated places (localities). The study team collected the legal data from January 11, 2023, through May 7, 2024. The sample included all US cities with more than 75 000 residents in 2016-2020 American Community Survey 5-year estimates and 3 census-designated places that met the population threshold and had local laws in place. To ensure that all states were represented, we additionally included the largest city for states that did not have a city with more than 75 000 residents. eFigure 1 in Supplement 1 provides a map and eTable 1 in Supplement 1 shows a list of the included localities. We followed the Strengthening the Reporting of Observational Studies in Epidemiology (STROBE) reporting guideline.^27^ The NYU Langone Institutional Review Board reviewed and approved the study as exempt under 45 CFR 46.104 category 4. The study was exempt because it was an analysis of publicly available secondary data or a secondary limited dataset containing only indirect identifiers where individual identities could not be reidentified.

### Legal Epidemiology Methods

We used legal epidemiology methods^28^ to classify syringe distribution laws at the state and local levels as of December 31, 2022. A detailed codebook guided data extraction and coding. As described elsewhere,^29^ state laws were identified in the legal database Westlaw Next and were extracted and coded by 2 lawyers (C.S.D. and A.D.).

Local laws were identified in municipal law databases and through manual searches of municipality websites. Because county authorization is required for SSPs to operate in Florida, we conducted an additional search for relevant laws in Florida counties following the same approach as the municipal laws. Laws were extracted and coded by nonlawyer team members (P.S., J.D., M.C.) with supervision from lawyer team members (C.S.D. and A.D.). For accuracy, 100% of laws were checked by the legal team. MonQcle^30^ was used to record data and manage legal data extraction.

### Exposure

The exposure was a cross-sectional measure of the combined state and local policy environment governing the legality of syringe distribution by SSPs as of December 31, 2022. Syringe distribution policy environments were grouped into 6 categories (Table 1).

**Table 1.** State and Local Policy Environments Governing the Legality of Syringe Distribution by SSPs as of December 31, 2022

State and local syringe distribution policy (N = 482)	Description
State prohibition (most restrictive) (n = 99)	Free syringe distribution is prohibited by state law with no exceptions for SSPs
Local prohibition (n = 14)	State does not prohibit syringe distribution or prohibits it with an exception for SSPs, but the locality prohibits syringe distribution without exception
Locality explicitly prohibits SSPs or does not authorize when required (n = 10)	State prohibits syringe distribution with an exception for legal SSPs, but locality prohibits SSPs specifically or lacks local SSP authorization required by state law
SSPs exempted from prohibition (n = 273)	Syringe distribution is generally prohibited by state or local law, but distribution via SSPs is permitted either generally or via specific authorization
No state or local prohibitions (least restrictive) (n = 78)	Syringe distribution is not prohibited by state or local law
Unknown (n = 8)	Syringe distribution is generally prohibited by state law with an exception for locally authorized SSPs, and local authorization status is unknown

Abbreviation: SSP, syringe services program.

### Outcome

The outcomes of interest were the binary presence (any vs 0 SSP locations) and the total number of publicly listed SSP locations (referred to herein as SSPs). To generate a dataset for this analysis, we relied on 2 data streams. First, we used the North American Syringe Exchange Network (NASEN) website which contains a directory of SSPs that agree to be publicly listed online. We manually extracted the names and all service location addresses for SSPs on the website that stated they distributed syringes as of February 2, 2024 (n = 1165 SSPs).^31^ We used Geocodio for geocoding latitude, longitude, and to obtain the Place FIPS code. Second, we conducted an independent search for state directories of SSPs. To do so, we used Google and iteratively searched for “[STATE] and syringe services,” “[STATE] and SSP,” and “[STATE] and needle exchange” for each of the 50 US states and the District of Columbia. We then reviewed results for directories or compilations of SSPs maintained or displayed on official state websites. Such directories were present on the websites in 24 unique states. Each list was downloaded for review and SSP address data were extracted for all service locations where SSPs were listed as operating. A combined list of directory-listed SSPs (N = 1095) was created. We used Geocodio for geocoding latitude, longitude, and to obtain the place Federal Information Processing Series (FIPS) code. Next, we compared the state directory and NASEN-generated lists and removed duplicates. Duplicates were identified based on SSP locations with matching addresses and a manual assessment for each SSP locations that was within 300 miles of another unique location. The deduplicated list contained a total of 1458 SSPs across the US. Finally, we restricted the list to the subset of 482 cities included in the study sample using place FIPS codes. This yielded a final sample of 654 SSPs. Another application of the same underlying data source appears in Joshi et al.^32^

In both data streams, we counted SSPs with multiple addresses at each location to most comprehensively measure SSP access. SSPs that reported only post office box locations were counted once in the locality of the listed address. SSPs that reported only a county or counties served and did not report an identifiable address or city were not assigned to a specific locality and were excluded from the analysis. Notably, while we aimed to be as comprehensive as possible, SSPs that do not publicly disclose their address or location online are not included in the outcome dataset. Therefore, we refer to SSPs included in this study as “publicly listed SSPs.”

### Covariates

Covariates were selected based on hypothesized relationships with the policy exposure and SSP presence. For each locality, we identified the political party affiliation of the state governor (or District of Columbia mayor; as of January 1, 2022),^33^ the presence or absence of a state overdose Good Samaritan law that provides protection from drug paraphernalia law arrests (as of December 31, 2021),^34^ and the state rate of new HIV cases (2022).^35^ We included the county fatal drug overdose rate per 100 000 residents (2022) from the National Vital Statistics System restricted-use files identified with underlying causes *International Classification of Diseases, Tenth Revision* (*ICD-10*), codes X40-44, X60-64, X85, and Y10-14.^36^ Localities that span multiple counties were assigned the median overdose mortality rate of the counties they fell within. At the local level, we included population size (log), proportion of residents aged 25 to 44 years (log) given that HIV transmission is highest among people aged 25 to 34 years^37^ and overdose mortality risk is highest among people aged 35 to 44 years,^38^ and proportion with a bachelor’s degree or higher (log), which was found to be associated with SSP presence,^39^ from American Community Survey 5-year estimates for 2018 through 2022.

### Statistical Analysis

First, we descriptively examined publicly listed SSP counts and covariate distribution across policy categories and localities with vs without any publicly listed SSP. Next, we used 2-part negative binomial hurdle models to examine the association between syringe distribution laws and the presence of any publicly listed SSP (binary outcome) and, for places with at least 1 publicly listed SSP, the association between policies and the total number of SSPs (count outcome). Localities classified as “unclear” (n = 8) and “locality explicitly prohibits SSPs or does not authorize when required” (n = 10) were excluded from the negative binomial hurdle model due to lack of clarity and variation in the outcome (ie, all had 0 SSPs), yielding an analytic sample of 464 localities. We assumed a negative binomial outcome distribution as there was evidence of outcome overdispersion (mean < variance) and an adjusted negative binomial model outperformed a Poisson model (based on the Akaike information criterion [AIC]). Given the high proportion of localities without any publicly listed SSPs (eFigure 2 in Supplement 1), we considered 0-inflated negative binomial and negative binomial hurdle modeling approaches; AIC values were similar between the 2 approaches. We selected a negative binomial hurdle approach because it allowed us to separately analyze policy associations with at least 1 SSP being present in a locality, vs the total number of SSPs among localities with at least 1 SSP. Policies most conducive to at least 1 SSP vs a greater number of SSPs may differ. For example, an analysis of a California state law that explicitly authorized SSPs found that the law was associated with a 46% increase in SSPs at the county level but identified a single case where a new SSP opened in a county that had 0 SSPs before the law change.^20^ This modeling approach enabled us to assess these different policy-relevant outcomes separately. We implemented hurdle models in R using the *pscl* package, version 4.5.2 (R Foundation for Statistical Computing); parameters were estimated by maximum likelihood.^40^ We included all defined covariates in both the binomial and count portions of the model; population (log) was included as an offset in the count portion of the model. We report the adjusted odds ratios (ORs) and 95% CIs of having any SSP from the binomial portion of the model, and the adjusted rate ratios (RRs) and 95% CIs of SSP counts among localities with at least 1 SSP from the truncated negative binomial portion of the model. The 95% CIs that did not contain the null value were considered statistically significant. Based on both binomial and count portions of the model, we predicted the total SSP count for each locality and obtained the average and 95% CI for each policy exposure category with the R package modelbased.^41^

For sensitivity analysis, first, we conducted a basic quantitative bias analysis to examine the level of differential misclassification of the outcome needed to invalidate the extension of the present findings to all SSPs, whether publicly listed online or not.^42^ We expect that SSPs in the most restrictive policy environments are least likely to identify publicly online. Therefore, we determined the percentage of SSPs in the state prohibition category classified as having 0 SSPs that would need to be reclassified as having 1 or more SSPs for the unadjusted OR between the policies of interest and binary SSP presence to have an OR equal to 1. Second, to examine the influence of outliers, we removed localities with an SSP count at or above the 99th percentile (>25 SSPs; n = 2 localities excluded) and reran the main analysis.

## Results

### SSP Syringe Distribution Legal Landscape

As of December 31, 2022, state law prohibited syringe distribution in 21% of 482 localities. Local law prohibited SSP syringe distribution in 3% of localities and explicitly prohibited SSPs or did not authorize SSPs when required in 2% of localities. State law, local law, or both exempted SSPs from a syringe distribution prohibition in 57% of localities. No state or local law prohibited syringe distribution in 16% of localities.

Most localities in the state prohibition policy category were located in the South (61%), followed by the Midwest (30%). Localities in the local prohibition policy category were mostly located in the Midwest (50%), followed by the South (29%). Localities where SSPs were exempt from prohibition were largely located in the West (60%), followed by the South (30%), while localities with no state or local prohibition policy category were mostly located in the Northeast (44%), followed by the West (28%) (eTable 2 in Supplement 1).

### SSP Outcome Distribution, 2024

Table 2 shows the distribution of the policy categories and covariates by publicly listed SSP presence. Overall, 38% of localities had at least 1 publicly listed SSP. Among localities with at least 1 publicly listed SSP (n = 181 [37.6%]), there was a mean (SD) of 3.6 (6.6) SSPs and a median of 2 SSPs (IQR, 1-3 SSPs). The least restrictive policy category (no state or local prohibitions on SSP syringe distribution) had the highest percentage of localities with any publicly identified SSP (63%), followed by the SSP exempted from the prohibition policy category (43%), the local prohibition policy category (29%), and the state prohibition policy category (12%).

**Table 2.** Distribution of Policy and Geographic Characteristics by Binary Presence of a Publicly Listed SSP

Characteristic	Any publicly listed SSP (n = 181 [37.6%])	No publicly listed SSP (n = 301 [62.4%])	Overall (N = 482 [100%])
Syringe distribution policy category, No. (row %)			
State prohibition	12 (12)	87 (88)	99 (100)
Local prohibition	4 (29)	10 (71)	14 (100)
Locality explicitly prohibits SSPs or does not authorize when necessary	0	10 (100)	10 (100)
Unknown	0	8 (100)	8 (100)
SSPs exempted from prohibition	117 (43)	156 (57)	273 (100)
No state or local prohibition	49 (63)	29 (37)	78 (100)
Publicly listed SSP counts			
Mean (SD)	3.6 (6.6)	NA	1.4 (4.4)
Median (IQR)	2 (1-3)	NA	0 (0-1)
Region, No. (row %)			
Midwest	35 (45)	42 (55)	77 (100)
Northeast	33 (69)	15 (31)	48 (100)
South	45 (27)	119 (73)	164 (100)
West	69 (36)	124 (64)	193 (100)
Locality population total, mean (SD)	353 411 (748 969)	151 691 (172 928)	227 860 (489 141)
Locality proportion aged 25-44 y, mean proportion (SD)	0.30 (0.04)	0.28 (0.03)	0.29 (0.03)
Locality proportion with Bachelor’s degree or higher, mean proportion (SD)	0.36 (0.14)	0.37 (0.15)	0.37 (0.15)
County all-drug overdose rate, per 100 000 population, mean (SD)	41.5 (23.7)	28.7 (14.5)	33.5 (19.5)
State new HIV diagnosis rate, per 100 000 population, mean (SD)	11.8 (5.4)	14.6 (5.7)	13.5 (5.7)
State governor, No. (row %)			
Democratic	124 (47)	140 (53)	264 (100)
Republican	58 (27)	160 (73)	218 (100)
State overdose Good Samaritan law with arrest protection, No. (row %)			
Yes	96 (36)	169 (64)	265 (100)
No	86 (40)	131 (60)	217 (100)

Abbreviations: NA, not applicable; SSP, syringe services program.

### Negative Binomial Hurdle Model Results

The adjusted odds ratio (OR) for localities with no state or local syringe distribution prohibition having any publicly listed SSP relative to state prohibition was 16.3 (95% CI, 6.0-44.1) (Table 3). The adjusted OR for localities with SSPs exempted from prohibition having any publicly listed SSP relative to state prohibition was 7.1 (95% CI, 2.9-17.6). There was no significant difference between the local prohibition and state prohibition categories.

**Table 3.** Adjusted Negative Binomial Hurdle Model Results, N = 464 Localities^a^

Estimate	Binomial portion of model, adjusted OR (95% CI)	Count portion of model, adjusted RR (95% CI)
Intercept	0.0 (0.0-0.0)	0.0 (0.0-0.0)
Policy category (reference: state prohibition)		
Local prohibition	2.9 (0.7-13.0)	3.1 (0.6-15.8)
SSPs exempted from prohibition	7.1 (2.9-17.6)	2.4 (1.1-5.6)
No state or local prohibition	16.3 (6.0-44.1)	2.7 (1.1-6.4)
Local and count covariates		
Local population total (log)	4.2 (2.7-6.6)	NA
Local proportion college (log)	1.0 (0.6-1.7)	1.7 (1.0-3.0)
Local proportion aged 25 to 44 y (log)	3.4 (0.4-33.4)	1.2 (0.2-7.6)
County all-drug-overdose mortality rate	1.0 (1.0-1.1)	1.0 (1.0-1.0)
State covariates		
State GSL has arrest protection (reference: state does not)	0.6 (0.3-1.1)	0.9 (0.6-1.4)
State new HIV diagnoses rate	0.9 (0.9-1.0)	1.0 (0.9-1.0)
State governor political party: Republican (reference: Democratic)	0.4 (0.2-0.7)	0.7 (0.5-1.1)

Abbreviations: GSL, Good Samaritan law; NA, not applicable; OR, odds ratio; RR, rate ratio; SSP, syringe services program.

^a^ Population (log) included as an offset in the count portion of the model (not shown).

Among localities with at least 1 publicly listed SSP, the adjusted rate ratio (RR) for localities with no state or local syringe distribution prohibition compared with the rate of the state prohibition policy category was 2.7 (95% CI, 1.1-6.4). The adjusted RR for localities with SSPs exempted from prohibition compared with the rate of the state prohibition policy category was 2.4 (95% CI, 1.1-5.6). There was no significant difference between the local prohibition and state prohibition categories. The highest predicted counts were in localities where SSPs were exempted from prohibition (predicted 1.9 publicly listed SSPs; 95% CI, 1.5-2.4) and localities with no state or local prohibition (predicted 1.5 publicly listed SSPs; 95% CI, 1.1-1.9) (Figure; eTable 3 in Supplement 1).

**Figure.**
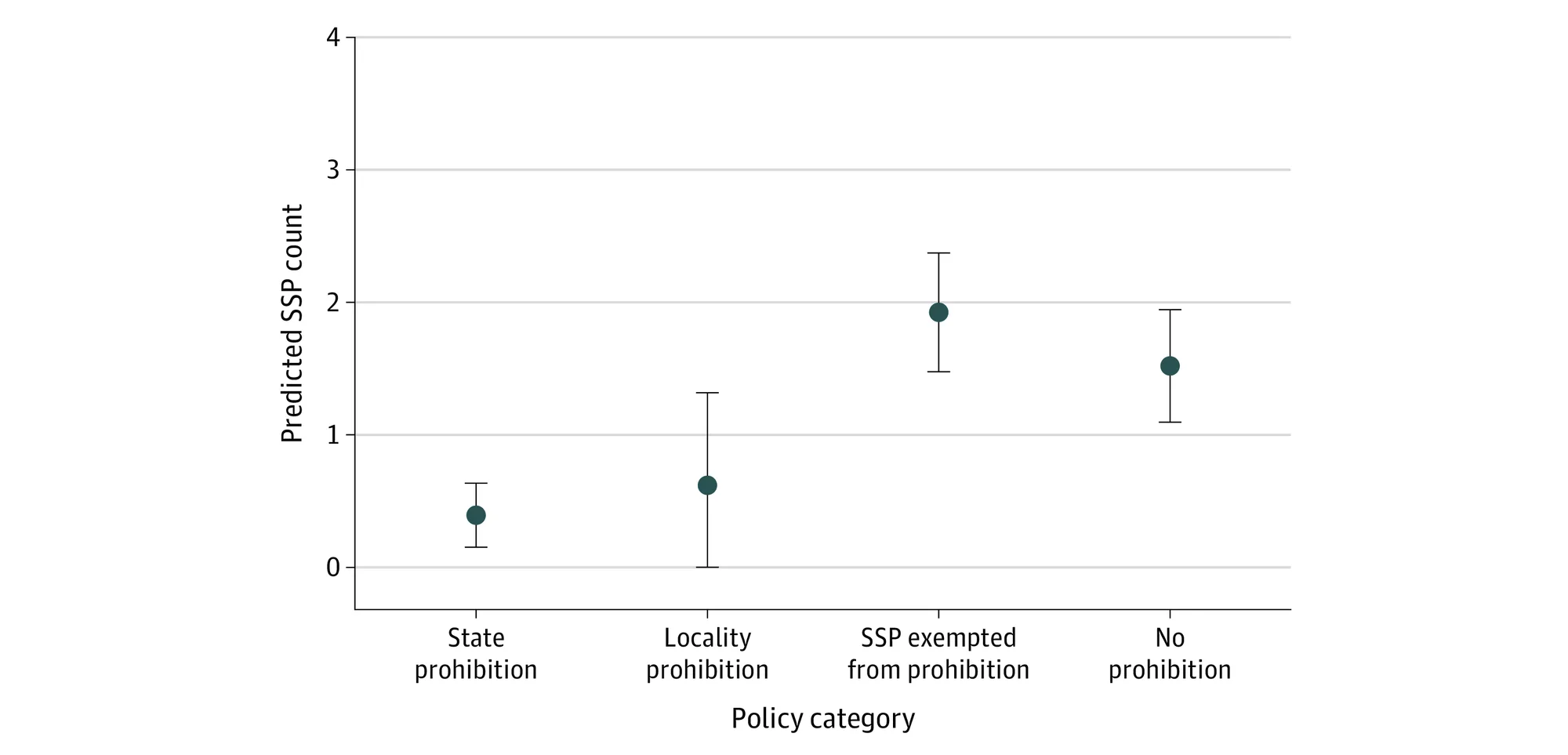
Dot Plot Showing the Predicted SSP Count in Localities by Policy Category, 2024 Predicted counts and 95% CIs of SSPs using both the binomial and count portions of the adjusted negative binomial hurdle model are shown. SSP indicates syringe services program.

### Sensitivity Results

Quantitative bias analyses indicated that, for localities with SSPs exempted from prohibition (unadjusted OR, 5.4) to have no association with the presence of any SSP, we would have had to misclassify approximately 35% of localities classified in the state prohibition policy category with no SSP presence. For places with no state or local prohibition (unadjusted OR, 12.3), we would have had to misclassify approximately 58% of these localities. The second sensitivity analysis suggested that the main adjusted OR and RR findings were robust to the exclusion of localities with more than 25 SSPs each (eTable 4 in Supplement 1).

## Discussion

More than 60% of the largest US cities did not have a publicly listed SSP as of February 2024. While there is an expectation and some evidence that the local legal environment is associated with SSP presence, this has not been quantitatively tested. Our results found that, across a national sample of US cities, the state and local SSP syringe distribution policy environment is an important factor in publicly listed SSPs’ presence and the total number of listed SSPs among localities with at least 1 SSP. Localities with no state or local syringe distribution prohibitions were the most likely to have a publicly listed SSP. The magnitude of the OR between policy categories and the presence of any publicly listed SSP was greater than the RR between policy categories and the total number of SSPs in localities with at least 1 SSP. This may suggest that state and local syringe distribution policies are a particularly salient variable in publicly listed SSPs operating, but once there is a publicly listed SSP present in a locality, the syringe distribution policy context may be a relatively less important factor for facilitating operation of additional SSPs. The lowest concentration of publicly reported SSPs was found in localities within states that prohibited all free syringe distribution.

There were regional differences in the distribution of policy contexts. Most localities in the most restrictive policy context were in the South. As southern states currently experience the highest burden of HIV,^43^ our findings suggest that restrictive syringe distribution policy contexts may be a structural factor that inhibits the availability of an evidence-based intervention to reduce that burden. There were also descriptive differences by region for the most prevalent policy context in localities without any publicly listed SSP. While most localities without SSPs in the Midwest and South were restricted by either a state or local syringe distribution prohibition, relatively few localities in the Northeast and West without an SSP were restricted by these prohibitions. Thus, factors external to the syringe distribution policy environment may be a more salient barrier to SSP presence in northeastern and western communities.

Notably, even in localities with less restrictive policy environments, the number of publicly listed SSPs was low. There was a median of 2 listed SSPs in localities with at least 1 SSP. This is an important consideration as sufficient access to SSPs is critical for maximum impact. There is substantial evidence that living in closer proximity to an SSP is associated with greater engagement and less injection equipment sharing^44,45,46^; thus, low SSP counts may not meet local needs. Additional work to understand the factors that drive growth in the number of operational SSPs is needed. The literature suggests that a lack of funding^21,47,48,49,50^ continues to pose a major barrier to SSP growth. These funding barriers likely contributed to the relatively low number of listed SSPs observed, including in areas with less restrictive policy contexts.

### Limitations

This study has limitations. First, this is a cross-sectional study that examined whether laws, as written, were associated with publicly listed SSP presence. The extent to which laws are implemented and enforced is not characterized, and there is evidence that legal protections for SSP participants are not always upheld by law enforcement officers.^51,52^ Second, we examined the legality of SSP syringe distribution; a wider range of laws may impact SSP presence and operations (eg, drug checking, naloxone access, syringe possession). Third, findings reflect the association between policy contexts and SSPs that are publicly listed online. We did not capture SSPs that were not publicly identified in NASEN or on a state directory in the outcome dataset. Thus, our SSP indicators (binary and count) reflect publicly listed SSPs and likely underestimate the true number of localities with any SSP and the true number of SSPs operating in our sample. However, our quantitative bias analysis provides a high level of support on our findings despite the data limitations. For our findings to be null, we would have needed to misclassify more than 58% of cities in the most restrictive policy exposure category that are currently classified as not having any SSP (state prohibition) and more than 35% of cities in the second most restrictive policy exposure category (SSPs exempted from prohibition). Fourth, we rely on binary and count indicators of publicly listed SSPs as the outcomes of interest; however, this does not account for the proximity of residents to a given SSP, nor does it capture whether SSPs are operating with fidelity^22^ to core components (eg, an example of core components of SSPs can be found in the study by Batty et al^53^) or factors that may impact access to services, such as funding levels and hours of operation. These factors may be critical for downstream population health impacts. Fifth, due to the policy exposure data available for analysis, the study was limited to a sample of the largest US cities. Thus, while findings are meaningful for larger population areas, they may not extend to rural settings; further work is necessary to better understand policy conditions most conducive to SSPs in rural locations.

## Conclusions

This study found more than 60% of the largest US cities did not have a publicly listed SSP as of February 2024, despite evidence that SSPs are critical interventions to reduce injection-related bloodborne disease transmission and increase access to other risk-reduction services. State and local syringe distribution laws were associated with the presence of any publicly reported SSP and were associated with the total number of SSPs in localities with at least 1 SSP. These results suggest that legal reform may represent an important lever for expanding access to harm-reduction services, particularly in regions with limited SSP availability. However, ensuring adequate SSP coverage may also require complementary strategies addressing funding, service delivery, and local implementation barriers.

## Notes

Supplement 1. **eFigure 1.** Map of U.S. geographic areas in study sample (n=482) **eTable 1.** List of geographic areas included in study sample (n=482) **eTable 2.** Distribution of geographic characteristics by syringe distribution policy category (n=482) localities **eFigure 2.** Publicly listed SSP count outcome histogram, n=482 localities **eTable 3.** Predicted Publicly Listed SSP counts from both portions of negative binomial hurdle models **eTable 4.** Adjusted negative binomial hurdle model sensitivity results dropping outliers, n=462 localities

Supplement 2.
Data Sharing Statement
