# Supplementary material for: State and Local Syringe Distribution Laws and the Presence of Syringe Services Programs
Source: JAMA Health Forum. 2026 Oct 23;7(10):e263730. doi: 10.1001/jamahealthforum.2026.3730 (PMC13695068; doi:10.1001/jamahealthforum.2026.3730)
Supplement: Supplementary file 1 [file jamahealthforum-e263730-s001.pdf]

# Supplemental Online Content

Doonan SM, Joshi S, Davis CS, et al. State and local syringe distribution laws and the presence of syringe services programs. *JAMA Health Forum*. 2026;7(10):e263730. doi:10.1001/jamahealthforum.2026.3730

**eFigure 1.** Map of U.S. geographic areas in study sample (n=482)
**eTable 1.** List of geographic areas included in study sample (n=482)
**eTable 2.** Distribution of geographic characteristics by syringe distribution policy category (n=482) localities
**eFigure 2.** Publicly listed SSP count outcome histogram, n=482 localities
**eTable 3.** Predicted Publicly Listed SSP counts from both portions of negative binomial hurdle models
**eTable 4.** Adjusted negative binomial hurdle model sensitivity results dropping outliers, n=462 localities

This supplemental material has been provided by the authors to give readers additional information about their work.

© 2026 Doonan SM et al. *JAMA Health Forum*.

eFigure 1. Map of U.S. geographic areas in study sample (n=482)

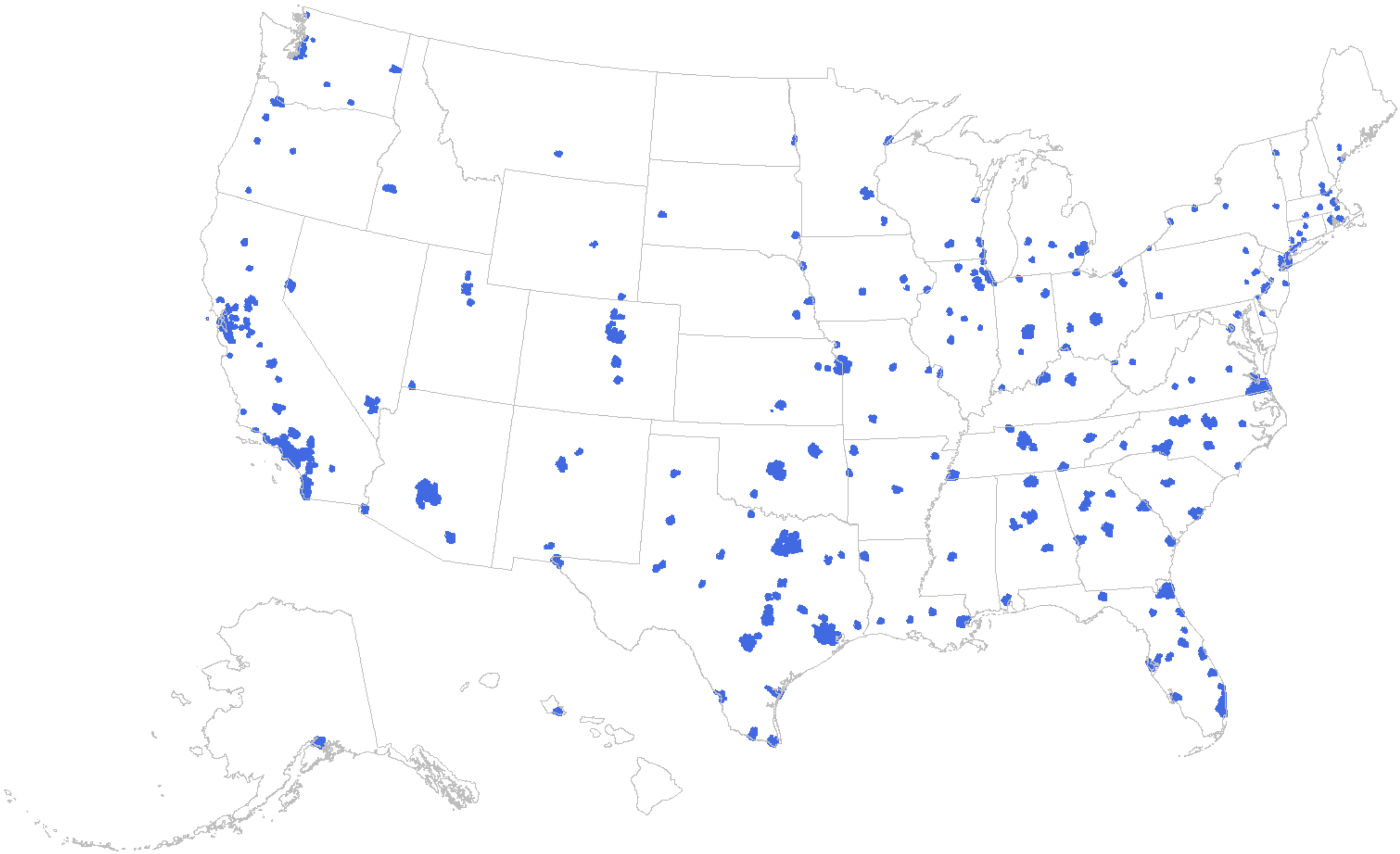

© 2026 Doonan SM et al. *JAMA Health Forum*.

eTable 1. List of geographic areas included in study sample (n=482)

| **Locality** | **State** |
|---|---|
| Birmingham | AL |
| Hoover city | AL |
| Huntsville city | AL |
| Mobile | AL |
| Montgomery | AL |
| Tuscaloosa | AL |
| Anchorage | AK |
| Avondale | AZ |
| Chandler | AZ |
| Gilbert | AZ |
| Glendale | AZ |
| Goodyear | AZ |
| Mesa | AZ |
| Peoria | AZ |
| Phoenix | AZ |
| Scottsdale | AZ |
| Surprise | AZ |
| Tempe | AZ |
| Tucson | AZ |
| Yuma city | AZ |
| Fayetteville | AR |
| Fort Smith | AR |
| Jonesboro | AR |
| Little Rock | AR |
| Springdale | AR |
| Alameda | CA |
| Alhambra | CA |
| Anaheim | CA |
| Antioch | CA |
| Bakersfield | CA |
| Baldwin Park | CA |
| Bellflower | CA |
| Berkeley | CA |
| Buena Park | CA |
| Burbank | CA |
| Carlsbad | CA |
| Carson | CA |
| Chico | CA |

© 2026 Doonan SM et al. *JAMA Health Forum*.

| | |
|---|---|
| Chino | CA |
| Chino Hills | CA |
| Chula Vista | CA |
| Citrus Heights | CA |
| Clovis | CA |
| Compton | CA |
| Concord | CA |
| Corona | CA |
| Costa Mesa | CA |
| Daly City | CA |
| Downey | CA |
| El Cajon | CA |
| Elk Grove | CA |
| El Monte | CA |
| Escondido | CA |
| Fairfield | CA |
| Folsom | CA |
| Fontana | CA |
| Fremont | CA |
| Fresno | CA |
| Fullerton | CA |
| Garden Grove | CA |
| Glendale | CA |
| Hawthorne | CA |
| Hayward | CA |
| Hemet | CA |
| Hesperia | CA |
| Huntington Beach | CA |
| Indio | CA |
| Inglewood | CA |
| Irvine | CA |
| Jurupa Valley | CA |
| Lake Forest | CA |
| Lakewood | CA |
| Lancaster | CA |
| Livermore | CA |
| Long Beach | CA |
| Los Angeles | CA |
| Manteca | CA |
| Menifee | CA |
| Merced | CA |

© 2026 Doonan SM et al. *JAMA Health Forum*.

| | |
|---|---|
| Milpitas | CA |
| Mission Viejo | CA |
| Modesto | CA |
| Moreno | CA |
| Mountain View | CA |
| Murrieta | CA |
| Napa | CA |
| Newport Beach | CA |
| Norwalk | CA |
| Oakland | CA |
| Oceanside | CA |
| Ontario | CA |
| Orange | CA |
| Oxnard | CA |
| Palmdale | CA |
| Pasadena | CA |
| Perris | CA |
| Pleasanton | CA |
| Pomona | CA |
| Rancho Cucamonga | CA |
| Redding | CA |
| Redwood City | CA |
| Rialto | CA |
| Richmond | CA |
| Riverside | CA |
| Roseville | CA |
| Sacramento | CA |
| Salinas | CA |
| San Bernardino | CA |
| San Buenaventura (Ventura) | CA |
| San Diego | CA |
| San Francisco | CA |
| San Jose | CA |
| San Leandro | CA |
| San Marcos | CA |
| San Mateo | CA |
| San Ramon | CA |
| Santa Ana | CA |
| Santa Barbara | CA |
| Santa Clara | CA |
| Santa Clarita | CA |

© 2026 Doonan SM et al. *JAMA Health Forum*.

| | |
|---|---|
| Santa Maria | CA |
| Santa Monica | CA |
| Santa Rosa | CA |
| Simi Valley | CA |
| South Gate | CA |
| Stockton | CA |
| Sunnyvale | CA |
| Temecula | CA |
| Thousand Oaks | CA |
| Torrance | CA |
| Tracy | CA |
| Tustin | CA |
| Union City | CA |
| Upland | CA |
| Vacaville | CA |
| Vallejo | CA |
| Victorville | CA |
| Visalia | CA |
| Vista | CA |
| West Covina | CA |
| Westminster | CA |
| Whittier | CA |
| Arvada | CO |
| Aurora | CO |
| Boulder | CO |
| Centennial | CO |
| Colorado Springs | CO |
| Denver | CO |
| Fort Collins | CO |
| Greeley city | CO |
| Lakewood | CO |
| Longmont | CO |
| Loveland | CO |
| Pueblo | CO |
| Thornton | CO |
| Westminster | CO |
| Bridgeport | CT |
| Danbury | CT |
| Hartford | CT |
| New Haven | CT |
| Norwalk | CT |

© 2026 Doonan SM et al. *JAMA Health Forum*.

| | |
|---|---|
| Stamford | CT |
| Waterbury | CT |
| Dover | DE |
| Wilmington | DE |
| Washington | DC |
| Boca Raton | FL |
| Boynton Beach | FL |
| Cape Coral | FL |
| Clearwater | FL |
| Coral Springs | FL |
| Davie town | FL |
| Deerfield Beach | FL |
| Deltona | FL |
| Fort Lauderdale | FL |
| Fort Myers | FL |
| Gainesville | FL |
| Hialeah | FL |
| Hollywood | FL |
| Jacksonville | FL |
| Lakeland | FL |
| Largo | FL |
| Melbourne | FL |
| Miami Beach | FL |
| Miami | FL |
| Miami Gardens | FL |
| Miramar | FL |
| Orlando | FL |
| Palm Bay | FL |
| Palm Coast | FL |
| Pembroke Pines | FL |
| Plantation | FL |
| Pompano | FL |
| Port St. Lucie | FL |
| St. Petersburg | FL |
| Sunrise | FL |
| Tallahassee | FL |
| Tampa | FL |
| West Palm Beach | FL |
| Athens-Clarke County | GA |
| Atlanta | GA |
| Augusta-Richmond County | GA |

© 2026 Doonan SM et al. *JAMA Health Forum*.

| | |
|---|---|
| Columbus | GA |
| Johns Creek | GA |
| Macon-Bibb County | GA |
| Roswell | GA |
| Sandy Springs | GA |
| Savannah | GA |
| South Fulton | GA |
| Warner Robins | GA |
| Urban Honolulu CDP | HI |
| Boise City | ID |
| Meridian | ID |
| Nampa | ID |
| Arlington Heights village | IL |
| Aurora | IL |
| Bloomington | IL |
| Champaign | IL |
| Chicago | IL |
| Cicero | IL |
| Elgin | IL |
| Joliet | IL |
| Naperville | IL |
| Peoria | IL |
| Rockford | IL |
| Springfield | IL |
| Waukegan | IL |
| Bloomington | IN |
| Carmel | IN |
| Evansville | IN |
| Fishers | IN |
| Fort Wayne | IN |
| Hammond | IN |
| Indianapolis | IN |
| South Bend | IN |
| Cedar Rapids | IA |
| Davenport | IA |
| Des Moines | IA |
| Iowa City | IA |
| Sioux City | IA |
| Kansas City | KS |
| Lawrence | KS |
| Olathe | KS |

© 2026 Doonan SM et al. *JAMA Health Forum*.

| | |
|---|---|
| Overland Park | KS |
| Topeka | KS |
| Wichita | KS |
| Lexington-Fayette county | KY |
| Louisville/Jefferson county | KY |
| Baton Rouge | LA |
| Lafayette city | LA |
| Lake Charles | LA |
| New Orleans | LA |
| Shreveport | LA |
| Lewiston | ME |
| Portland | ME |
| Baltimore | MD |
| Boston | MA |
| Brockton | MA |
| Cambridge | MA |
| Fall River | MA |
| Lawrence | MA |
| Lowell | MA |
| New Bedford | MA |
| Newton | MA |
| Somerville | MA |
| Springfield | MA |
| Worcester | MA |
| Ann Arbor | MI |
| Dearborn | MI |
| Detroit | MI |
| Farmington Hills | MI |
| Grand Rapids | MI |
| Kalamazoo | MI |
| Lansing | MI |
| Livonia | MI |
| Sterling Heights | MI |
| Troy | MI |
| Warren | MI |
| Westland | MI |
| Wyoming | MI |
| Bloomington | MN |
| Brooklyn Park | MN |
| Duluth city | MN |
| Minneapolis | MN |

© 2026 Doonan SM et al. *JAMA Health Forum*.

| | |
|---|---|
| Plymouth | MN |
| Rochester | MN |
| St. Paul | MN |
| Jackson | MS |
| Columbia | MO |
| Independence | MO |
| Kansas City | MO |
| Lee's Summit | MO |
| O'Fallon | MO |
| St. Joseph | MO |
| St. Louis | MO |
| Springfield | MO |
| Billings | MT |
| Lincoln | NE |
| Omaha | NE |
| Henderson | NV |
| Las Vegas | NV |
| North Las Vegas | NV |
| Reno | NV |
| Sparks | NV |
| Manchester | NH |
| Nashua | NH |
| Clifton | NJ |
| Elizabeth | NJ |
| Jersey City | NJ |
| Newark | NJ |
| Paterson | NJ |
| Toms River CDP | NJ |
| Trenton | NJ |
| Albuquerque | NM |
| Las Cruces | NM |
| Rio Rancho | NM |
| Santa Fe | NM |
| Albany | NY |
| Buffalo | NY |
| New Rochelle | NY |
| New York | NY |
| Rochester | NY |
| Syracuse | NY |
| Yonkers | NY |
| Asheville | NC |

© 2026 Doonan SM et al. *JAMA Health Forum*.

| | |
|---|---|
| Town of Carey | NC |
| Charlotte | NC |
| Concord | NC |
| Durham | NC |
| Fayetteville | NC |
| Gastonia | NC |
| Greensboro | NC |
| Greenville | NC |
| High Point city | NC |
| Raleigh | NC |
| Wilmington | NC |
| Winston-Salem | NC |
| Fargo | ND |
| Akron | OH |
| Cincinnati | OH |
| Cleveland | OH |
| Columbus | OH |
| Dayton | OH |
| Parma | OH |
| Toledo | OH |
| Broken Arrow | OK |
| Edmond | OK |
| Lawton | OK |
| Norman | OK |
| Oklahoma City | OK |
| Tulsa | OK |
| Beaverton | OR |
| Bend | OR |
| Eugene | OR |
| Gresham | OR |
| Hillsboro | OR |
| Medford | OR |
| Portland | OR |
| Salem | OR |
| Allentown | PA |
| Bethlehem | PA |
| Erie | PA |
| Philadelphia | PA |
| Pittsburgh | PA |
| Reading | PA |
| Scranton | PA |

© 2026 Doonan SM et al. *JAMA Health Forum*.

| | |
|---|---|
| Cranston | RI |
| Providence | RI |
| Warwick | RI |
| Charleston | SC |
| Columbia | SC |
| Mount Pleasant town | SC |
| North Charleston | SC |
| Rapid City | SD |
| Sioux Falls | SD |
| Chattanooga | TN |
| Clarksville | TN |
| Franklin | TN |
| Knoxville | TN |
| Memphis | TN |
| Murfreesboro | TN |
| Nashville-Davidson metropolitan government | TN |
| Abilene | TX |
| Allen | TX |
| Amarillo | TX |
| Arlington | TX |
| Austin | TX |
| Baytown | TX |
| Beaumont | TX |
| Brownsville | TX |
| Bryan | TX |
| Carrollton | TX |
| Cedar Park | TX |
| College Station | TX |
| Conroe | TX |
| Corpus Christi | TX |
| Dallas | TX |
| Denton | TX |
| Edinburg | TX |
| El Paso | TX |
| Flower Mound town | TX |
| Fort Worth | TX |
| Frisco | TX |
| Garland | TX |
| Georgetown | TX |
| Grand Prairie | TX |
| Houston | TX |

© 2026 Doonan SM et al. *JAMA Health Forum*.

| | |
|---|---|
| Irving | TX |
| Killeen | TX |
| Laredo | TX |
| League City | TX |
| Lewisville | TX |
| Longview | TX |
| Lubbock | TX |
| McAllen | TX |
| McKinney | TX |
| Mesquite | TX |
| Midland | TX |
| Mission | TX |
| Missouri City | TX |
| New Braunfels | TX |
| Odessa | TX |
| Pasadena | TX |
| Pearland | TX |
| Pharr | TX |
| Plano | TX |
| Richardson | TX |
| Round Rock | TX |
| San Angelo | TX |
| San Antonio | TX |
| Sugar Land | TX |
| Temple | TX |
| Tyler | TX |
| Waco | TX |
| Wichita Falls | TX |
| Layton | UT |
| Ogden | UT |
| Orem | UT |
| Provo | UT |
| St. George | UT |
| Salt Lake City | UT |
| Sandy | UT |
| West Jordan | UT |
| West Valley City | UT |
| Burlington city | VT |
| South Burlington city | VT |
| Alexandria | VA |
| Arlington CDP | VA |

© 2026 Doonan SM et al. *JAMA Health Forum*.

| | |
|---|---|
| Chesapeake | VA |
| Hampton | VA |
| Lynchburg | VA |
| Newport News | VA |
| Norfolk | VA |
| Portsmouth | VA |
| Richmond | VA |
| Roanoke | VA |
| Suffolk | VA |
| Virginia Beach | VA |
| Auburn | WA |
| Bellevue | WA |
| Bellingham | WA |
| Everett | WA |
| Federal Way | WA |
| Kennewick | WA |
| Kent | WA |
| Kirkland | WA |
| Renton | WA |
| Seattle | WA |
| Spokane | WA |
| Spokane Valley | WA |
| Tacoma | WA |
| Vancouver | WA |
| Yakima | WA |
| Charleston | WV |
| Huntington | WV |
| Green Bay | WI |
| Kenosha | WI |
| Madison | WI |
| Milwaukee | WI |
| Racine | WI |
| Casper | WY |
| Cheyenne | WY |

© 2026 Doonan SM et al. *JAMA Health Forum*.

eTable 2. Distribution of geographic characteristics by syringe distribution policy category (n=482) localities

| | **State prohibits** (N=99) | **Locality prohibits** (N=14) | **Locality explicitly prohibits** (N=10) | **Unknown** (N=8) | **SSPs exempt-ed from prohib-ition** (N=273) | **No prohib-ition** (N=78) |
|---|---|---|---|---|---|---|
| **Any Publicly Listed SSP**, n (%) | 12 (12.1%) | 4 (28.6%) | 0 (0%) | 0 (0%) | 117 (42.9%) | 49 (62.8%) |
| **Total Publicly Listed SSPs,** mean (SD) | 0.374 (1.48) | 0.571 (1.16) | 0 (0) | 0 (0) | 1.81 (5.58) | 1.47 (2.11) |
| **Total Publicly Listed SSPs**, median [Min, Max] | 0 [0, 11] | 0 [0, 4] | 0 [0, 0] | 0 [0, 0] | 0 [0, 71] | 1 [0, 12] |
| **Region,** n (%) | | | | | | |
| Midwest | 30 (30.3%) | 7 (50.0%) | 0 (0%) | 6 (75.0%) | 21 (7.7%) | 13 (16.7%) |
| Northeast | 7 (7.1%) | 0 (0%) | 0 (0%) | 0 (0%) | 7 (2.6%) | 34 (43.6%) |
| South | 60 (60.6%) | 4 (28.6%) | 8 (80.0%) | 2 (25.0%) | 81 (29.7%) | 9 (11.5%) |
| West | 2 (2.0%) | 3 (21.4%) | 2 (20.0%) | 0 (0%) | 164 (60.1%) | 22 (28.2%) |
| **Governor: Republican**, n (%) | 79 (79.8%) | 3 (21.4%) | 8 (80.0%) | 6 (75.0%) | 88 (32.2%) | 34 (43.6%) |
| **State Overdose GSL with Arrest Protection**, n (%) | 11 (11.1%) | 6 (42.9%) | 10 (100%) | 5 (62.5%) | 205 (75.1%) | 28 (35.9%) |
| **State New HIV Diagnosis Rate**, per 100,000 population, mean (SD) | 14.5 (6.56) | 11.8 (5.39) | 20.6 (2.95) | 13.5 (5.29) | 14.5 (4.91) | 8.32 (4.18) |
| **County All-Drug Overdose Rate**, per 100,000 population, mean (SD) | 24.3 (18.1) | 39.9 (14.3) | 41.6 (14.1) | 34.9 (15.6) | 35.8 (20.0) | 34.9 (17.9) |
| **Locality Population Total**, rounded mean (SD) | 238,000 (339,000) | 126,000 (56,000) | 196,000 (267,000) | 97,600 (16,600) | 257,000 (609,000) | 150,000 (129,000) |
| **Locality Proportion Aged 25-44**, mean % (SD) | 0.28 (0.03) | 0.27 (0.02) | 0.26 (0.04) | 0.27 (0.01) | 0.29 (0.03) | 0.29 (0.04) |
| **Locality Proportion with Bachelor's degree or higher**, mean % (SD) | 0.36 (0.13) | 0.38 (0.16) | 0.30 (0.08) | 0.37 (0.21) | 0.37 (0.15) | 0.36 (0.16) |

© 2026 Doonan SM et al. *JAMA Health Forum*.

eFigure 2. Publicly listed SSP count outcome histogram, n=482 localities

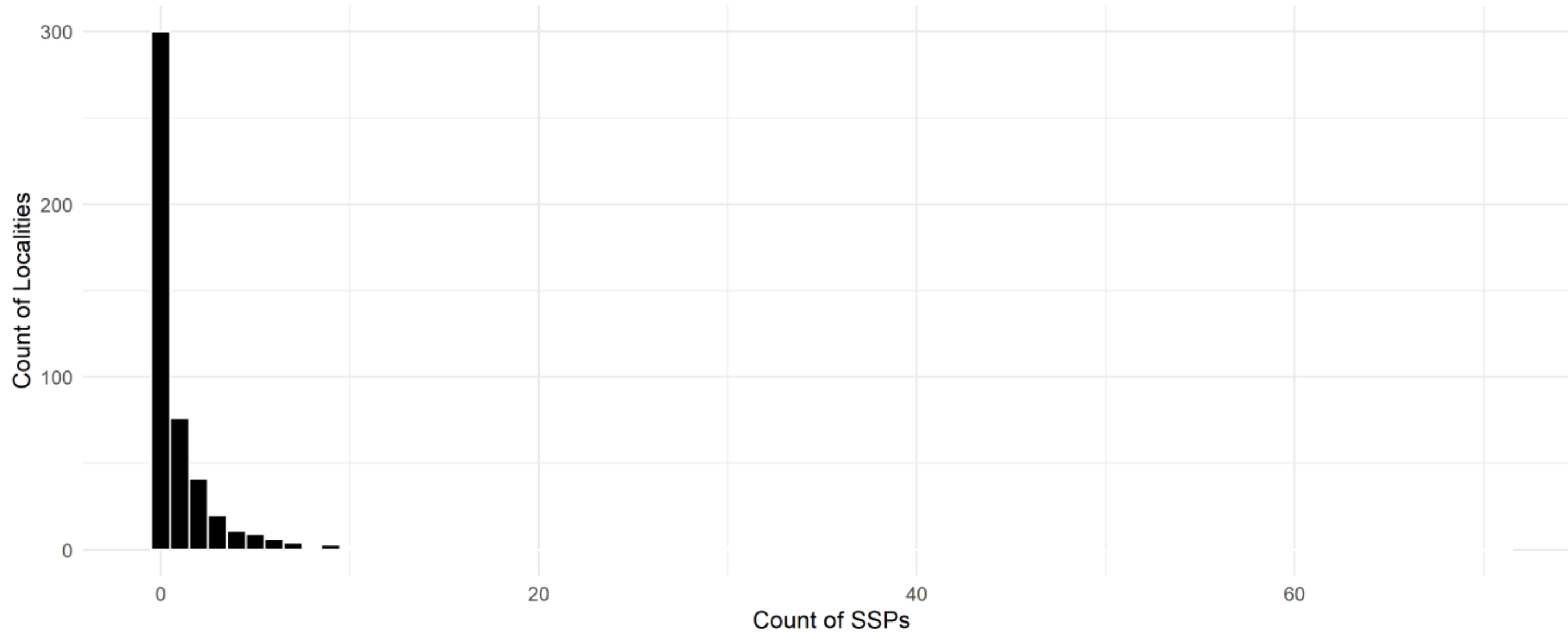


© 2026 Doonan SM et al. *JAMA Health Forum*.

eTable 3. Predicted Publicly Listed SSP counts from both portions of negative binomial hurdle models

| Policy | Predicted Publicly Listed SSP count | Lower 95% CI | Upper 95% CI |
|---|---|---|---|
| State prohibited | 0.4 | 0.2 | 0.6 |
| Locally prohibited | 0.6 | 0* | 1.3 |
| SSPs exempted from prohibition | 1.9 | 1.5 | 2.4 |
| No prohibitions | 1.5 | 1.1 | 1.9 |

Notes. Showing predicted counts of publicly listed SSPs using both the binomial and count portions of the adjusted negative binomial hurdle model. *Truncated at 0.

© 2026 Doonan SM et al. *JAMA Health Forum*.

eTable 4. Adjusted negative binomial hurdle model sensitivity results dropping outliers,

n=462 localities

| | Binomial portion of model | | | Count portion of model | | |
|---|---|---|---|---|---|---|
| | aOR | Lower 95% CI | Upper 95% CI | aRR | Lower 95% CI | Upper 95% CI |
| Intercept | 0.0 | 0.0 | 0.0 | 0.0 | 0.0 | 0.0 |
| Locally prohibited (ref=state prohibited) | 2.9 | 0.7 | 13.0 | 3.0 | 0.6 | 14.5 |
| SSPs exempted from prohibition | 7.1 | 2.9 | 17.5 | 2.1 | 0.9 | 4.7 |
| No prohibitions | 16.3 | 6.0 | 44.0 | 2.6 | 1.1 | 6.0 |
| Population total (log) | 4.2 | 2.7 | 6.6 | - | - | - |
| Proportion college (log) | 1.0 | 0.6 | 1.7 | 2.2 | 1.3 | 4.0 |
| State New HIV Diagnoses Rate | 0.9 | 0.9 | 1.0 | 1.0 | 0.9 | 1.0 |
| Local proportion aged 25 to 44 (log) | 3.4 | 0.4 | 33.5 | 0.9 | 0.2 | 5.6 |
| County all-drug overdose mortality rate | 1.0 | 1.0 | 1.1 | 1.0 | 1.0 | 1.0 |
| State GSL has arrest protection (Ref. state does not) | 0.6 | 0.3 | 1.1 | 1.0 | 0.7 | 1.6 |
| Governor is Republican (Ref. Governor is not) | 0.4 | 0.2 | 0.7 | 0.8 | 0.5 | 1.2 |

Note. Population (log) included as an offset in the count portion of the model (not shown).

© 2026 Doonan SM et al. *JAMA Health Forum*.
