# Supplementary material for: State and Local Syringe Distribution Laws and the Presence of Syringe Services Programs
Source: JAMA Health Forum. 2026 Oct 23;7(10):e263730. doi: 10.1001/jamahealthforum.2026.3730 (PMC13695068; doi:10.1001/jamahealthforum.2026.3730)
Supplement: Supplementary file 2 [file jamahealthforum-e263730-s002.pdf]

Data Sharing Statement

Doonan. State and Local Syringe Distribution Laws and the Presence of Syringe Services Programs. *JAMA Health Forum*. Published October 23, 2026.
doi:10.1001/jamahealthforum.2026.3730

Data
Data available: No

Additional Information
Explanation for why data not available: Legal policy data is available from the study team upon reasonable request.
